# The causal relationship between depression and frozen shoulder: A two-sample Mendelian randomization

**DOI:** 10.1097/MD.0000000000035556

**Published:** 2023-11-03

**Authors:** Guang-hua Deng, Yong-kang Wei

**Affiliations:** a Ya’an Hospital of Traditional Chinese Medicine; b The Fourth Clinical College of Xinjiang Medical University.

**Keywords:** depression, frozen shoulder, Mendelian randomization

## Abstract

To investigate the causal relationship between depression and frozen shoulder using a Mendelian randomization (MR) approach. Pooled data from a large-scale genome-wide association study were used. Genetic loci that were independent of each other and associated with depression and frozen shoulder in populations of European ancestry were selected as instrumental variables. Inverse variance weighting was used as the primary analysis method. Weighted median and MR-Egger were used as complementary analysis methods to assess causal effects. To explore the causal relationship between depression and frozen shoulder. Sensitivity test analysis was performed using heterogeneity test, multiple validity test, and leave-one-out analysis to explore the robustness of the results. Inverse variance weighting results showed an odds ratio (95% confidence interval) of 1.18 (0.91–1.53), *P* = .204, indicating that depression was not causally related to the development of frozen shoulder. And the test revealed no heterogeneity and pleiotropy, and the sensitivity analysis also showed robust results. In this study, genetic data were analyzed and explored using a two-sample MR analysis, and the results showed no causal relationship between depression and the occurrence of frozen shoulder, requiring the inclusion of a larger sample for the study.

## 1. Introduction

Frozen shoulder, also known as adhesive capsulitis,^[1]^ is a pathological condition characterized by pain and limited joint motion in the shoulder.^[2]^ There are usually no significant findings in the patient’s history, clinical examination, or imaging evaluation to explain the loss of motion or pain.^[3,4]^ Depression is a chronic disease^[5,6]^ with a high global prevalence, affecting 14% of men and 15% of women.^[7,8]^ Several retrospective and prospective studies to investigate the association between depression and a frozen shoulder have found that depression may be an important risk factor for a frozen shoulder, but the results have been inconsistent.^[9,10]^ Therefore, the causal relationship between depression and frozen shoulder still needs further investigation.

In traditional epidemiological research methods, the causal inferences obtained are considered to be of limited value because of the influence of confounding factors and reverse causality.^[11]^ In contrast, Mendelian randomization (MR), a genetic epidemiological method, is a useful tool to assess the causal role of depression and frozen shoulder.^[12]^ By using genetic variants such as single nucleotide polymorphism (SNP) as instrumental variables that can modify disease risk factors or exposures, MR studies can strengthen causal inferences about exposure-outcome associations.^[13]^ According to Mendel’s law of inheritance, genetic variants are not susceptible to confounding factors because they are randomly assigned during gamete formation.^[14]^ In addition, confounding factors and reverse causality correlations can be minimized because genotypes cannot change with disease progression.^[15]^

To this end, we conducted a two-sample MR study to examine the association of genetic susceptibility to depression with frozen shoulder risk factors. We aimed to provide important evidence for the causal role of depression in causing a frozen shoulder.

## 2. Data and Methods

### 2.1. Data sources

The largest sample size of genome-wide association study (GWAS) data for depression and frozen shoulder was obtained through the IEU OpenGWAS project (mr cieu. ac. uk). The website was accessed on 2023-06-06. The final population source for all data was Europe, of either sex. Including depression (finn-b-F5_DEPRESSIO) containing 16,380,457 SNPs, 23,424 in the observation group and 192,220 in the control group; frozen shoulder (ebi-a-GCST90000512) containing 15,184,371 SNPs with a sample size of 451,099.This study was a re-analysis of previously collected and published data and therefore did not require additional ethical approval.

### 2.2. Conditioning of SNP as an instrumental variable

First, the instrumental variables were highly correlated with exposure, with *F* > 10 as a strong correlation criterion.^[16]^ Secondly, the instrumental variable was not directly correlated with the outcome, but only influenced the outcome through exposure, meaning that there was no genetic pleiotropy. In this study, the MR-Egger regression model with a non-zero intercept term (*P* < .05) indicated the absence of genetic pleiotropy.^[17]^ Third, instrumental variables were not related to unmeasured confounding.^[18]^ Finally, the human genotype-phenotype association database Phenoscanner V2 was searched for phenotypes associated with instrumental variables at genome-wide significance levels to determine whether these SNPs were associated with potential risk factors.^[19]^

### 2.3. SNP screening

Significant SNPs were screened from the GWAS summary data of depression (*P* < 5 × 10^−7^ was used as the screening condition)^[20]^; the linkage disequilibrium coefficient *r*^2^ was set to 0.001 and the width of the linkage disequilibrium region was 10,000 kb to ensure that each SNP was independent of each other.^[21]^ The above-screened depression-related SNPs were extracted from the GWAS summary data of frozen shoulder, while SNPs directly related to the outcome index were excluded (*P* < .05). The *F* value of each SNP was calculated, and SNPs with weak instrumental variables (*F* value less than 10) were excluded.^[22]^ And the human genotype-phenotype association database was queried to screen for potentially associated risk factor SNPs and to exclude them.^[23]^

### 2.4. Causality validation methods

The causal relationship between exposure (depression) and outcome (frozen shoulder) was verified mainly using inverse variance weighting (IVW), supplemented by weighted median (WME) and MR-Egger MR analysis methods, using SNPs as instrumental variables.

### 2.5. Sensitivity analysis

Various methods were used for sensitivity analysis. First, the Cochran *Q* test was used to assess the heterogeneity among the individual SNP estimates, and a statistically significant Cochran *Q* test proved that the analysis was significantly heterogeneous. Second, the MR pleiotropy residual sum and outlier (MRPRESSO) was used to validate the results in the IVW model, correct for the effects of outliers, and if outliers existed, they were excluded and the analysis was repeated. Third, the MR-Egger intercept test was used to test the horizontal multiplicity of SNPs. If the intercept term in the MR-Egger intercept test analysis was statistically significant, it indicated that the MR analysis had significant horizontal multiplicity. Fourth, leave-one-out analyses were performed by removing a single SNP at a time to assess whether the variation drove the association between the exposure and outcome variables. Fifth, funnel plots and forest plots were constructed to visualize the results of sensitivity analyses. *P* < .05 suggests a potential causal relationship for MR analysis and is statistically significant. All statistical analyses were performed using the “TwoSampleMR” package in R software version 4.3.0.

## 3. Results

### 3.1. Instrumental variables

Three SNPs that were strongly associated with depression (*P* < 5 × 10^−7^) without linkage disequilibrium (*r*^2^ < 0.001, kb = 10,000) were screened in this study. Three SNPs remained by taking the intersection with SNPs from the GWAS pooled data of depression while excluding SNPs directly associated with outcome indicators. In our study, each SNP had an *F* value greater than 10, indicating no weak instrumental variables (Table 1). We searched the human genotype-phenotype association database and no potentially associated risk factor SNPs were found.

**Table 1:** Information on the final screening of depression SNPs from GWAS data (n = 3).

ID	SNP	Effect_Allele	Other_Allele	β	SE	*P*	*F*
1	rs113392839	A	G	0.3299	0.0632	1.76E-07	27
2	rs113661867	T	C	0.1389	0.0272	3.25E-07	26
3	rs9809577	A	C	−0.0787	0.0143	3.32E-08	30

GWAS = genome-wide association study, SE = standard error, SNP = single nucleotide polymorphism.

### 3.2. Causal relationship between depression and frozen shoulder

**Figure 1.**
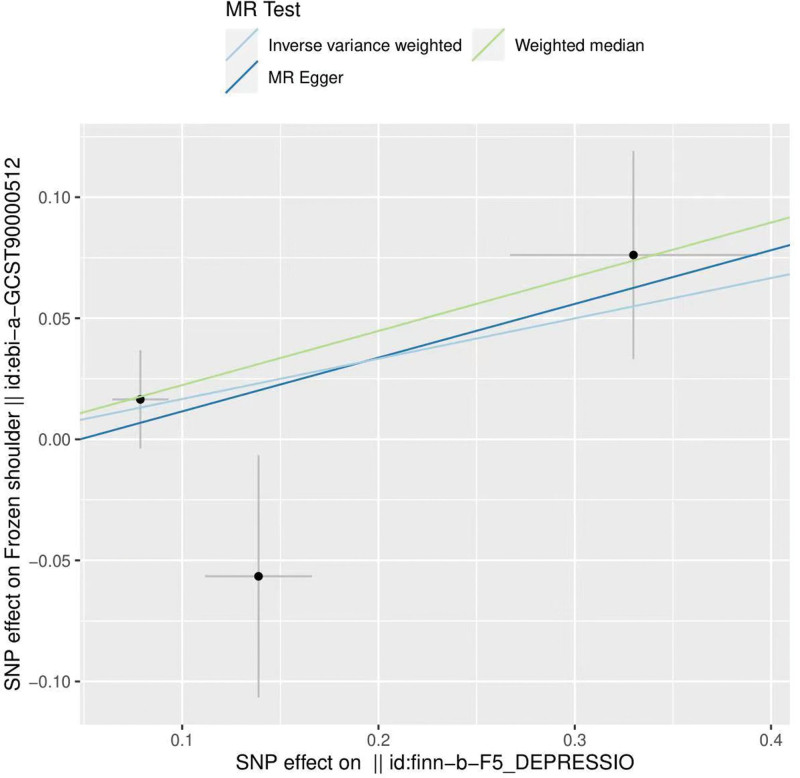
Scatter plot of depression and frozen shoulder.

**Figure 2.**
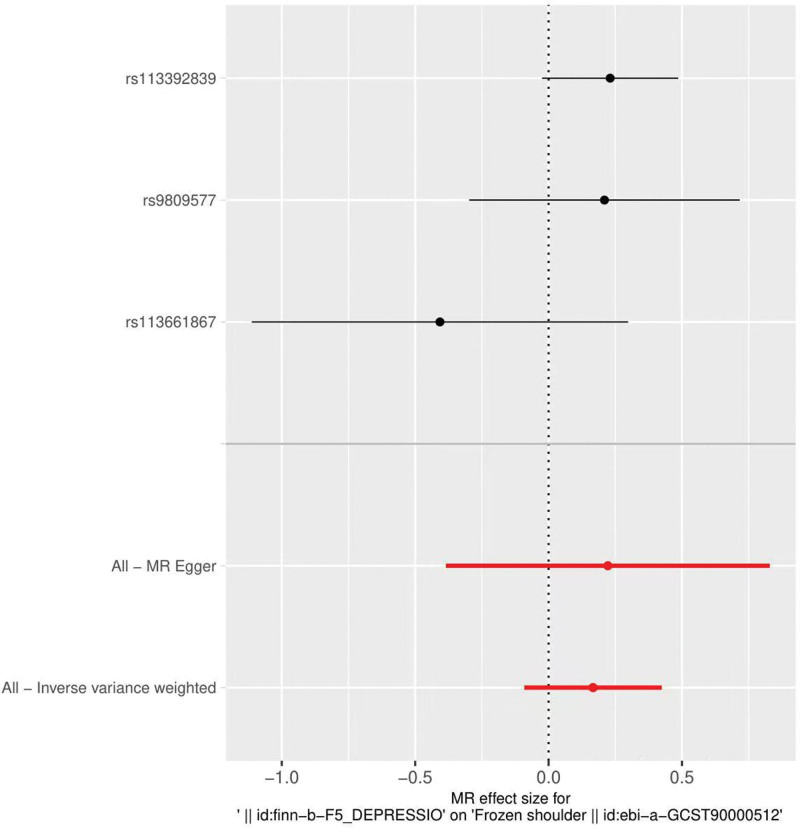
Forest plot of depression and frozen shoulder.

By MR analysis, the results of all 3 MRs, IVW, WME, and MR-Egger, showed a positive association between depression and frozen shoulder (Fig. 1). However, none of the differences were statistically significant, meaning that there was no causal relationship between depression and frozen shoulder (Fig. 2). IVW: odds ratio (OR) = 1.18, 95% confidence interval (CI) = 0.91–1.53, *P* = .204; WME: OR = 1.25, 95% CI = 0.97–1.61, *P* = .082; MR Egger: OR = 1.25, 95% CI = 0.68–2.29, *P* = .604 (Table 2).

**Table 2:** MR regression results of the 3 methods.

Method	β	SE	OR (95% CI)	*P*
IVW	0.166	0.131	1.18 (0.91–1.53)	.204
WME	0.223	0.129	1.25 (0.97–1.61)	.082
MR-Egger	0.221	0.309	1.25 (0.68–2.29)	.604

CI = confidence interval, IVW = inverse variance weighting, MR = Mendelian randomization, OR = odds ratio, SE = standard error, WME = Weighted median Egger.

### 3.3. Sensitivity analysis

**Figure 3.**
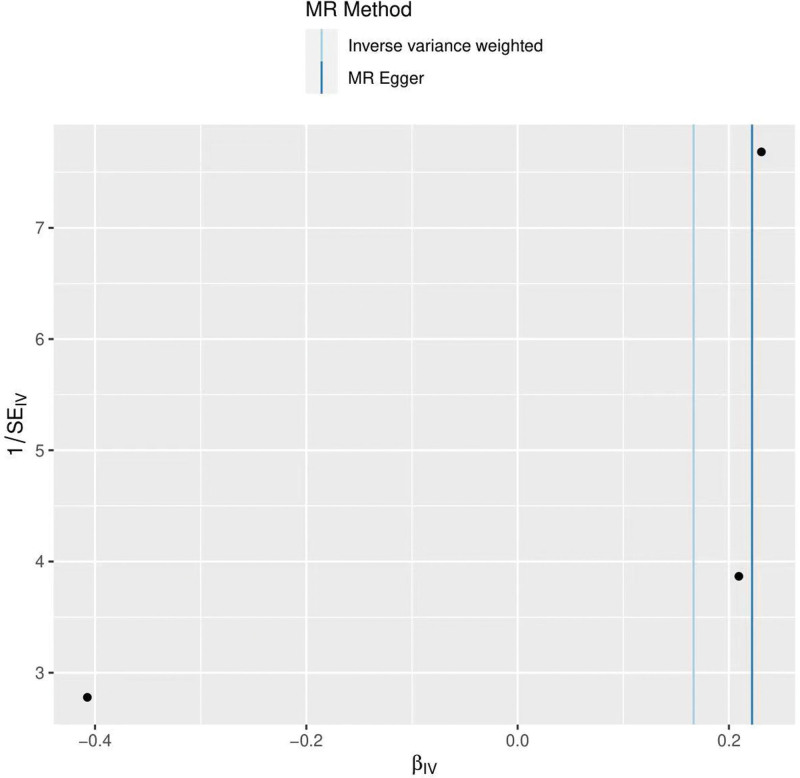
Funnel plot of depression and frozen shoulder.

**Figure 4.**
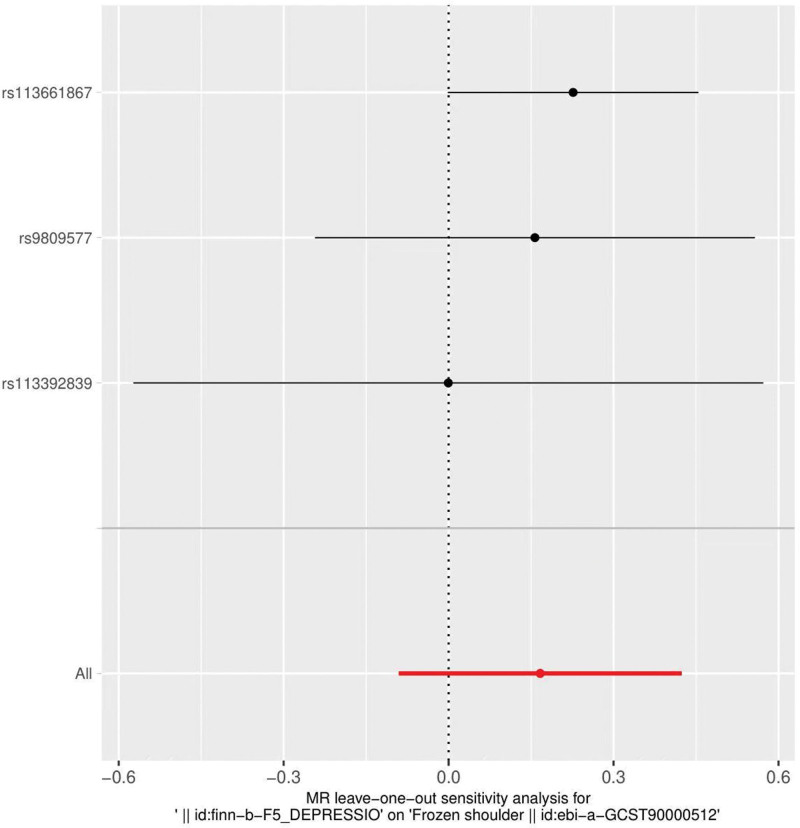
Analysis of depression and frozen shoulder by the leave-one-out method.

The heterogeneity test (Cochran *Q* test, *P* = .245) was performed using the IVW method and the results suggested that there was no heterogeneity. A funnel plot was drawn to show the heterogeneity results, as shown in Figure 3. MR-PRESSO was used to screen for SNPs that might cause heterogeneity, and no SNPs were found to cause heterogeneity in the results. The results of the global test by MR-PRESSO suggested that there was no pleiotropy (*P* = .862). The IVW method was used by default for the leave-one-out method, and as seen in Figure 4, the results of the remaining SNPs after eliminating any of them were on the right side of the valid line, indicating that the results were robust.

## 4. Discussion

It is known that depression may be a risk factor for frozen shoulder, but the causality of this association is unclear. Our MR study aimed to reveal a causal relationship between depression and a frozen shoulder. As shown by the two-sample MR results, the results showed no causal relationship between depression and the occurrence of a frozen shoulder, with an OR (95% CI) of 1.18 (0.91–1.53), *P* = .204.

Ding et al studied anxiety and depression in 124 patients (78 women and 46 men) with primary frozen shoulder and compared them with 130 (72 women and 58 men) age-, sex-, and education-matched healthy controls, and found that the prevalence of depression and anxiety was 24.2% and 6.41%, respectively, in patients with frozen shoulder, with a significantly higher prevalence than in the non-diseased population.^[9]^ Louis et al retrospectively studied the relationship between patients with frozen shoulder and depression in Germany, and after propensity matching analysis, a total of 29,258 patients with and 29,258 patients without frozen shoulder were included in the study, through which 17.5% of patients with and 8.7% of patients without frozen shoulder were found to suffer from depression (*P* < .001). This result was confirmed in a Cox regression analysis, as there was a pos**itive** and significant association between frozen shoulder and the cumulative incidence of depression (HR = 1.86, 95% CI: 1.78–1.95).^[24]^ By retrospectively analyzing 1983 patients with frozen shoulder during the novel coronavirus epidemic, Mello et al found a significantly increased risk of 8.8-fold (*P* < .001) of developing frozen shoulder in depressed patients.^[25]^

However, Floren et al prospectively included 77 patients who underwent arthroscopic rotator cuff repair. At 6-month follow-up, eight patients were diagnosed with frozen shoulder (group A) and 65 patients had a satisfactory range of motion of the joint (group B), and by comparing the 2 groups depression was found not to be a risk factor for the development of frozen shoulder after rotator cuff repair.^[10]^

The present study confirmed the absence of a causal relationship between depression and the development of frozen shoulder from a genetic perspective. The results of the current study differ from the findings of previous retrospective studies. Previous retrospective studies have concluded that depression increases the prevalence of frozen shoulders, but the results of the present study showed that depression does not increase the prevalence of frozen shoulders. However, the results were consistent with the results of the prospective study by Floren.^[10]^ The causal inferences obtained may be considered to be of limited value because retrospective studies are susceptible to confounding factors and reverse causality. In contrast, MR analysis is a new epidemiological approach that uses genetic variation as an instrumental variable of exposure to enhance causal inferences. This approach reduces the effects caused by confounding factors.^[26]^

At the same time, this study has some limitations. First, since all data were obtained from a population of European ancestry, the results do not represent a truly random population sample and are not applicable to other so races. Second, although various sensitivity analyses have been performed in this study to test the hypotheses of the MR study, it is also difficult to completely rule out a horizontal multiplicity of instrumental variables. Finally, the current sample size of GWAS data is still not large enough, and more in-depth studies using more GWAS data are needed in the future.

## 5. Conclusion

In conclusion, this study used a two-sample MR analysis to analyze and explore the genetic data, and the results showed no causal relationship between depression and the occurrence of frozen shoulder, and more samples need to be included in the study.

## Author contributions

**Conceptualization:** Yong-kang wei.

**Data curation:** Guang-hua deng.

**Formal analysis:** Guang-hua deng.

**Investigation:** Guang-hua deng.

**Methodology:** Guang-hua deng.

**Project administration:** Guang-hua deng.

**Resources:** Guang-hua deng.

**Software:** Guang-hua deng.

**Supervision:** Guang-hua deng.

**Validation:** Guang-hua deng.

**Visualization:** Guang-hua deng.

**Writing – original draft:** Guang-hua deng.

**Writing – review & editing:** Yong-kang wei.
